# The Pathology and Treatment of Yellow Fever; with Some Remarks upon the Nature of Its Cause and Its Prevention

**Published:** 1881-11

**Authors:** H. D. Schmidt

**Affiliations:** New Orleans, La.


### ARTICLE IV.

### THE PATHOLOGY AND TREATMENT OF YELLOW FEVER; WITH SOME REMARKS UPON THE NATURE OF ITS CAUSE AND ITS PREVENTION. By H. D. Schmidt, M.D., New Orleans, La. (Concluded from page 394, October No., 1881.)

Aside from the medicines above mentioned there will, in the great majority of cases, be no further demand for others during the remaining part of the course of the disease; henceforth, the physician must concentrate his whole attention upon the study of the clinical phenomena of the case, and upon the close superintendence of the nursing of the patient; and, to accomplish this task properly, he must not limit himself to general directions, but, moreover, explain to the friends or nurse the importance of closely watching the case. He must, furthermore, take the trouble of *giving them a detailed account of the phenomena which might manifest themselves during his absence*, and what should be done in such event to meet them before he is able to see the patient himself. Yellow fever, as we have seen, is a disease of a rapid course, in which the gravest pathological changes may take place in the space of only a few days, and, if anything at all can safely be done to ameliorate the noxious effects of the poison, it must be done during the febrile stage of the disease.

With the progressive rise of the fever the patient becomes more restless, particularly from the sensation of the increased amount of heat retained within the organism, while at the same time a delirium will gradually set in, slight at first, but generally increasing in violence with the progress of the disease. It is hardly necessary to repeat that the appearance of the delirium indicates the abnormal afflux of blood to the head, and the beginning of the hyperæmia of the brain, which, if passing beyond certain limits, will necessarily cause the death of the patient. Therefore, as soon as the temperature of the scalp commences to rise, applications of cold water should be made to the whole cranium. A towel, twice folded to obtain four layers, and *large enough to cover the whole head*, saturated with ice-water, will answer every purpose. These applications, if conscientiously made and attended to, I regard as the most important point in the whole treatment of the disease, though, if *irregularly* and *carelessly* applied, they will fail in their purpose. In order to avoid all interruption in their applications, there should be two towels in use, one of which to be in the ice-water always ready for application, as soon as the other is removed from the head. The most convenient method for keeping them always cold is to put a large piece of ice in a wash-basin containing just enough water to submerge the towel. In many cases it will be found that the heat of the head is so great as to warm the cold cloth in a few minutes. Though it is a difficult task to keep the head of the patient covered with the wet cloth while he is in a highly restless condition, even laboring under delirium, tossing his head from side to side, and frequently changing his position, yet *it must be done*, for if omitted during the *height* of the delirium, even for a short time, it may be followed by fatal consequences. It is most important, therefore, that at this period, the patient should never be left alone. The efficacy of these cold applications is proven by the patient becoming more quiet every time a fresh cloth is applied, and by his asking for their renewal, whenever he is in a state of consciousness—even after the febrile stage is over. Great care must be taken in making these applications not *too cold*, which would give rise to the opposite condition of the hyperæmia, a depression of the functions of the brain, no less dangerous than the congestion. For this reason, ice should not be applied directly to the head. To the extent of my observation, ice-water, if properly applied, answers all purposes.

While these ice-water applications serve to keep the hyperæmia of the brain within safe limits, they at the same time reduce the temperature of the body by abstracting a considerable amount of heat by way of the head. With the view of lowering the temperature of the body, it is recommended by many physicians to sponge with cold water, alcohol or any other rapidly evaporating liquid. As I regard the perspiration of the skin as one of the processes by which the poison is eliminated from the body, I have never adopted this practice for fear of putting a check to this process, which itself, by the evaporation taking place, can only promote the discharge of heat from the body. Besides, by indiscriminately exposing the surface of the body to the air, difficult to avoid, the blood-vessels of the skin are apt to contract suddenly and *unevenly*, driving the blood to some interior organ of the body, and increasing there the already existing congestion. It appears to me, therefore, that a cold bath of short duration would even be safer to the patient than the imperfect and unequal abstraction of heat by sponging, as the former, by acting more evenly upon the whole surface of the body, would not only more equally abstract the heat, but also prove a stimulant to the nervous system, especially if followed by a timely reaction of the skin.

For a number of years, now, the cold bath has been very extensively used in febrile diseases, especially in Germany, for the purpose of reducing the temperature of the body. This treatment is based upon the theory that the fatality of the case depends upon the abnormally augmented heat, giving rise to the disintegration of the albuminous substances, and, moreover, causing the various parenchymatous degenerations. Although the statistics taken from a large number of cases, in which the cold bath was used, compared with others of antecedent times and without the use of the bath, evidently show a considerable decrease in the mortality of these diseases, the theory of the fatality of the case depending upon the high temperature is by no means generally adopted. On the contrary, the beneficial effects of the cold bath are by many pathologists attributed to the stimulation which it affords to the nervous system; and this appears to be the true explanation.

"*Warfwinge* * of Kopenhagen, who not long ago investigated this subject more closely, expresses the opinion that the dangerous influence of a high temperature upon the tissues, and particularly upon the muscular element of the heart, has never been proved, and that, accordingly, the high temperature in infectious diseases is of little significance; but that the real danger consisted in an excessive formation of the specific poison of the disease. The collected 2,239 cases of typhus fever, of which 357, or minus the complicated cases, 230 terminated fatally. Only in one-eighth of these cases the temperature rose in the last days to 40° C. or higher; in the remaining seven-eighths the temperature was 39 to 40, 38 to 39, or even under 38° C. In considering the earlier stages of the disease it was found that in 52.7 per cent. of the fatal cases the temperature never reached 40°, and in two-thirds of the remaining percentage of the latter it was not higher than 40 to 40.4° only in one-sixth 41° were reached or passed. From this Warfwinge concluded that in typhus fever the degree of the degenerate processes in the tissues, as well as the derangement of the functions of the organs and the nervous system, are not at all proportionate to the height of the temperature. And, as in his opinion, it is not proved that the temperature in the interior of the body can be lowered by cold bath, he furthermore concluded that the cold baths exerted but a small influence upon the course of the disease, and should chiefly be regarded as a stimulus for the nervous system."

Besides, there are other diseases and conditions of the organism, such as tetanus, in which the temperature of the body reaches a very high degree, without promoting the disintegration of the tissues or inducing the parenchymatous degenerations of the organs. Even in febrile diseases the disintegration of the albuminous substances commences, as indicated by the augmentation of urea in the urine, before the elevation of temperature, as has been mentioned before.

Nevertheless, cold baths of *short duration*, and repeated at intervals of time during the hot stage, may prove beneficial in the treatment of yellow fever, if used only for the purpose of

* *Virchow u. Hirsch*, Jahresbericht Jour. das Jahr, 1878. Vol. I, p. 205.

stimulating and equalizing the functions of the nervous system; though I think that they require, in order to render them safe to the patient, more care and attention than could be generally bestowed upon the patients during an extensive epidemic, when trusty nurses are comparatively scarce. The manner in which they should be given I will quote from Liebermeister,* who has used them very extensively. It is as follows: "For adult patients the full length cold bath of 68° F., or lower, is to be preferred. The same water can be used for several successive baths for the same patient; the bath-tub remains standing full, and the water, representing about the temperature of the room, answers the purpose without change. The duration of the bath should be about ten minutes. If prolonged beyond that it becomes unpleasant to the patient, and may even prove a damage to him. If feeble persons are much affected by the bath, remaining cold and collapsed for a long time, the duration should be reduced to seven, or even to five minutes. A short cold bath like this will have a much better effect than a longer one of lukewarm water. Immediately after the bath the patient should have rest; he is therefore to be wrapped up in a dry sheet and put to bed (which may with advantage be warmed, especially at the foot), lightly covered, and given a glass of wine. In dealing with very feeble patients, one may begin with baths of a higher temperature, say 75°, although of course these will produce less effect. A method, especially recommended in such cases, if the surroundings permit, is that recommended by Ziemssen, of baths gradually cooled down, beginning with about 95°, and adding cold water gradually until the temperature is reduced to 72°, or below. These baths should be of longer duration."

"As a rule, in somewhat severe cases, I have the temperature taken every two hours day and night. Whenever the temperature in the rectum reaches 103°, or in the axilla 102.2°, a cold bath is given. As a matter of course, however, individual peculiarities must be taken into consideration. In children, or in persons whom one has reason to suppose capable of great resistance to the influence of heat, the temperature which calls for the bath may be placed higher, say 104° in the rectum, or 103° in

* *Ziemssen*, Cyclopædia of the Practice of Medicine. Amer. edition, vol. I, p. 208.

the axilla. In those, on the contrary, with less than the average resisting power, it may be well to employ the bath before so high a temperature has been reached, and according to the circumstances of the case, give a shorter bath, or a warmer one, or the gradually reduced bath of Ziemssen."

In following the above directions for the application of the cold bath in febrile diseases, however, it should be remembered that they were written in a cold, rigorous climate, like that of Germany; some allowance should therefore be made, on the other hand, for the semi-tropical one under which we live, where most of the people are accustomed to a certain amount of continuous heat of the weather almost throughout all seasons of the year.

It is hardly necessary to pass any remarks on the method of reducing the temperature of the body by permanently placing the patient upon a cot for the purpose of keeping up a continuous spray of cold water over the whole body,—the impracticability of which the inventor proved by paying with his own life for the unphilosophical idea of preventing the perspiration of the skin, and inducing a depression of the nervous system.

As regards the delirium I have to add that, in the epidemic of 1867, I have, in severe cases, frequently made use of a fly-blister to the nape of the neck, leaving it long enough to produce suppuration. The object, as may be guessed, was to invite the blood from the congested brain to the sore. But, although this practice was generally followed by favorable results, I would not pretend to assert that they were solely due to the application of the blister, but rather suspect that the cold applications to the head, used at the same time, contributed their full share. In the succeeding epidemics I omitted the blister, and directed my attention chiefly to the proper application of the cold to the head, of which the results were equally favorable, and without giving so much trouble to the patient, as is always associated with the application of a blister in that locality.

The pain over the bladder, associated with a temporary retention of urine, frequently observed during the febrile stage, is generally relieved by the application of a warm flax-seed poultice with some laudanum poured over it, to be continued until the pain is relieved and the urine commences to flow.

The nausea and constant inclination to vomit during the febrile stage of the disease is very distressing to the patient, and difficult to allay. The application of a sinapism over the epigastrium affords sometimes relief, but not permanently. I have before remarked that the condition of the stomach is a sure index of the condition of the liver, and the latter of the condition of the whole organism; it is therefore important that the stomach should be examined from time to time, in order to ascertain whether it is sensitive to pressure upon it, or not. As long as there is no pain produced by the pressure there is no immediate danger to the patient, while, on the other hand, the production of pain by pressure indicates a highly congested condition of this organ and a considerable fatty infiltration of the liver, which possibly may be accompanied by a rupture of the minute vessels of the mucous membrane of the stomach, giving rise to black vomit. As soon, therefore, as the least tenderness is noticed, such measures as will induce a revulsion from that organ to the skin should at once be taken. In many cases a mustard cataplasm answers all purposes; in others, however, the emplast. cantharides must be resorted to.

In order to allay the thirst of the patient, small fragments of ice may be given to be dissolved in the mouth, or even to be swallowed entire; the moderate cold thus produced upon the mucous membrane of the stomach may contribute to restore to a certain degree the tonus of the minute blood vessels. The ice, however, must be given with discrimination, as an excessive cooling down of the stomach might at the same time exert a depressing influence upon the sympathetic ganglia of the solar plexus, situated in the close vicinity of this organ. The same danger will accompany the application of solid ice to the epigastrium for the purpose of arresting the black vomit. If the latter really occur, the most reliable remedy, perhaps, is the swallowing of small pieces of ice. Iced champagne is a favorite remedy with many physicians, and I have often ordered it myself, though I would not assert that it exerted a special influence upon the blood vessels of the stomach; if followed by good results it may be presumed that they were chiefly due to the ice.

If the patient has safely passed the febrile stage, accompanied by the hyperæmia of the brain, the chief danger during the second stage, especially if the delirium was severe, lies in the great depression of the nervous system, being mostly felt by the heart, but also by other organs, as the liver and kidneys. The depression of the heart's action, however, may be increased by the degeneration of a part of its muscular elements, occurring in severe cases, and which may lead to a paresis or even paralysis of this organ. For this reason, as soon as the febrile storm has passed, the action of the heart should be closely watched, and if found too weak for the performance of its function, stimulants should at once be administered. While some physicians make use of digitalis, carbonate of ammonia, etc., which may well answer the purpose, others, perhaps the greater number, prefer the alcoholic stimulants; for my own part, I have always used the latter, as they are generally ready at hand and answer the purpose equally as well. They must, however, be administered with great caution and discrimination, as by an excessive use they may do as much harm as good, by overstimulation being followed by a worse depression, or by irritating the stomach leading to black vomit. Unfortunately, they are but too frequently misapplied to the disadvantage of the patient; from all I know, brandy and whisky flow too freely during an epidemic of yellow fever, so much so, that the people have become accustomed to regard these liquids as the chief remedy for the disease. I have learned of cases in which they have actually been given during the febrile stage of the disease, when they obviously can only increase the already existing excitement and augment the disturbance of the functions of the organs. On the other hand, however, I have met with cases, in which a tablespoonful of pure whisky, slightly diluted with water, and administered at the proper time, has evidently saved the patient's life by arousing the very much depressed action of the heart. As regards the selection of the stimulants, it may be said that while the ordinary wines, besides containing too little alcohol, are unsuitable on account of their excessive acidity and impurity, the finer sorts, like genuine old Sherry and Madeira, are too expensive and, also, difficult to be obtained. Pure brandy or whisky, therefore, will be the most preferable stimulants.

If during the course of the disease the urinary discharges are interrupted, the catheter, of course, should be used, in order to ascertain whether there is any urine in the bladder, or whether the non-appearance of this fluid is really due to a deficiency of secretion, depending upon the degeneration of the epithelium of the uriniferous tubules or the obstructions caused by the various infarctions in the latter. In those cases in which a suppression of the urinary secretion really occurs, it appears to me that nothing direct or reliable could be done to induce the kidneys to resume their functions, as the interruption depends upon the organic changes in these organs; though, if depending merely upon the infarctions of the tubules, especially the straight, these obstructions may be overcome, in some cases, and cleared by the pressure of the urine from behind them; the urine, then, will re-appear. On the whole, the stimulation of the nervous system appears to me here the proper course to be pursued; though, in such cases, the condition of the organs in general is already so low as to leave but very small chances for the recovery of the patient.

As regards the diet in yellow fever it may be said that during the febrile stage of the disease it is of little importance, for the reason that the patient generally refuses anything in the shape of food offered to him. Nevertheless, it appears to me that the stomach should not be left entirely empty during the stage, and some light nourishment in liquid form should be given. A thin soup of barley, or any other farinaceous liquid answers perhaps the purpose best; and, if given cold, may at the same time serve to allay the thirst. In the second stage, small doses of the essence of beef—always freshly prepared—or milk will answer the purpose. It is sufficiently known that some caution should be observed during the convalescence in not over-feeding the patient, an error which may be followed by very serious consequences. The diet, therefore, should be chiefly albuminous, very digestible, and given in small doses at first, until the congestion of the stomach may have entirely disappeared. But, it is not only the stomach where the danger of over-feeding lies; for, though the food may be properly digested and the nutritive matters absorbed into the blood, the degenerated and weakened organs and tissues may not be able to assimilate them, and a new disturbance in their nutrition is apt to arise. Alcoholic stimulants also should be given in convalescence with considerable moderation; in most cases they are not needed at all, the proper nourishing food alone will be sufficient to restore the strength of the patient.

The above remarks may be regarded as mere outlines of a rational treatment of yellow fever, to be filled with the details by the good judgment of the practicing physician. A number of special medicines have been, besides, used in this disease by many physicians; but as they have not only failed to exert any influence upon the neutralization or elimination of the infectious poison, but perhaps rather done harm, I forbear to mention and discuss them. Nevertheless, I would by no means discourage the trial of certain remedies in this disease—empirical as the experiment may be—if administered upon some rational principle. For, if a specific remedy neutralizing the poison should ever be discovered, it will probably be by empirical experiment. Such experiments can only be advantageously made in a hospital, and by experienced physicians, accustomed to systematic scientific investigations; therefore, the general practitioner will do better by following the simple rules of treatment given above until some special and better method has been ascertained.

### REMARKS ON THE PREVENTION OF YELLOW FEVER.

Although the subject of the prevention of yellow fever has always received the attention of the medical profession, it has never agitated the public mind to such an extent as since the occurrence of the epidemic of 1878. Aside from the numerous articles which have been written on this subject, so much has been said in speeches and lectures delivered, that I cannot properly conclude this treatise without likewise offering some brief remarks on the prevention of this disease.

Though, from all that of late has been said and written on this subject, it might be presumed that the prevention of a contagious disease was a most difficult and complicated task, it will appear more simple when examined closer, and in a systematic manner and form. Neither is there any novelty in the attempt made for the purpose of preventing disease, for this subject has been studied for many years by the scientific men of all civilized nations, most particularly during the last fifteen years, since the occurrence of the cholera epidemic in Europe ; and the treatises and articles containing the results of the experiments and investigations relating to it are quite numerous, forming a considerable part of the medical literature of these years. Besides, many of these experiments have been made in so thorough a manner, that, without addition, they may equally serve as a safe basis in devising means for the prevention of yellow fever.

As a contagious disease can only be prevented by the destruction of its specific cause, it is obvious that the *true nature* of the latter must *first be ascertained*, before any reliable means could be devised to arrest its activity. Without this knowledge the means employed will be *empirical* in their application, and in most instances, *as experience has sufficiently shown*, be attended with *a failure* of the object in view. Thus, if the disease, as some suppose, is generated by the gases arising from decomposing animal and vegetable matter, the destructive agent, or so-called "disinfectant," should possess the property of neutralizing these gases ; or, if, according to another theory, it is produced by the presence of minute organisms contained in the air we breathe, the disinfectant should be destructive to the lives of these beings, and, accordingly, be brought into close contact with them ; or, again, if depending merely upon certain climatic or meteorological conditions, as some people suppose, there can be no hope for relief, unless the climate could be changed ; and, lastly, if propagated by morbid secretions derived from the diseased organism itself, the disinfectant should be capable of decomposing the poison.

Supposing the theory which teaches the origin of yellow fever from gases arising from decomposing animal and vegetable matter in the streets and houses of a city to be true, then the means of prevention would be quite simple, consisting in keeping the streets and the interior of houses clean from this matter, with fair prospects of being delivered from the yellow pestilence. But though clean streets and houses are very recommendable, and indicate a certain degree of civilization, this theory, as far as it concerns yellow fever, rests, for the reasons already given, upon a very weak foundation.

Closely allied to this theory is the "bilge-water" theory, according to which the cause of the disease is to be sought either in the gases arising from this liquid, or in the minute organisms which it contains. If this theory was as true as it is false, there would be no difficulty of keeping yellow fever from our shores, as there is no want of agents which would neutralize the gases, or kill the organism, in a circumscribed space like the hold of a ship, though the proposed freezing process, having no rational foundation, would, like other empirical experiments, prove a decided failure.

Greater difficulties, however, would be encountered in the prevention of yellow fever, if the disease depended upon the presence of minute organisms contained in the air and breathed by the people, as here, the only effectual mode of destroying the cause would consist in bringing the organisms into actual contact with some disinfectant agent, as, for instance, carbolic acid *in solution*. Accordingly, every little corner of the streets and houses, as well as the furniture, bedding and clothes of the people should be wetted with this solution, a task impossible to be accomplished. To use the disinfectant in a gaseous form would be equally as impracticable, for the reason that, in order to destroy the organisms, it would be necessary to use the gas in such a concentrated form as to cause the death of the surrounding people, before it would in the least affect the organisms. For ten successive years the streets of New Orleans have been sprinkled over with carbolic acid solution, without ever having made the slightest impression upon the cause of yellow fever. It must, therefore, have now become obvious that unless these or similar measures are carried out in a most thorough manner, it is useless to resort to them at all.

If yellow fever were really caused by noxious gases arising from animal and vegetable decomposition in the streets and houses, or by the bilge-water in the ships, or by bacteria or other minute organisms, the prospects of mitigating or preventing the disease would not be very encouraging, as dirt and filth will occur in any large city, and in defiance of the best regulated and executed sanitary laws. But, fortunately, this is not the case, as it is more probable that its true cause is a specific animal poison, similar in nature to that of small-pox, scarlet fever, or other contagious diseases, or to other animal and vegetable poisons,—substances, the noxious influence of which may, with the proper caution and attention, be counteracted, or, at least, to a certain degree limited.

In the preceding pages I have already sufficiently demonstrated the nature of the poison, and the manner in which it is propagated and carried from place to place, but have omitted to offer any remarks on the proper mode of prevention. In entering upon this subject it becomes necessary, first, to inquire in what manner the poison is probably introduced, or called into activity in a community. Thus, if yellow fever should appear at New Orleans, or any other seaport, *after having been entirely absent for several years*, it is very probable, or *almost certain*, that its poison has been imported anew, and most likely by ships hailing from infected ports. But, if the disease should re-appear in a place one or two years, or even longer, after having prevailed there, the probability arises that the poison is derived from the previous cases, having remained in the interval of time in a dormant (condensed) state, *i. e.*, for the want of the conditions necessary to its re-assuming the vaporous or gaseous form, as already explained. This probability, however, does not preclude a simultaneous fresh importation. While thus the poison may, on the one hand, be newly imported by ships, it may also lurk about from year to year in the same place, as is the case with small-pox, scarlet fever, measles, etc., the communicability of which nobody doubts. The extent to which the disease may spread, after having originated in this manner, cannot be determined, as it depends on various circumstances or so-called "chances." It is more than probable that those sporadic cases, occurring almost every year, and whose origin cannot be traced, arise from these sources; and it is certain that the yellow fever epidemic of 1879, in Memphis, originated from the poison which, by adhering to the old clothes, had over-wintered in the shoemaker's box. It will be remembered that in this epidemic the first cases occurred in one and the same neighborhood, and were confined to certain houses or families.

From the numerous experiments made relating to the nature of animal poisons or contagia and their disinfectants, almost every investigator has drawn the conclusion that the contagion will lose its noxious properties by a free exposure to the atmosphere, and that for this reason the latter, promoting the process of oxydation, is the best disinfectant; while, on the other hand, the poison will retain its properties when kept in a place from which the atmosphere and light are excluded. Now, it is well known that after an epidemic of yellow fever, the majority of people in whose houses cases and deaths have occurred, *do not destroy or even properly disinfect* the bedding or other articles to which the poison emanating from the patients has adhered; but that, on the contrary, especially among the lower walks of life, these articles are left as they were, and, in many instances, kept in dark and badly ventilated places, where there is no opportunity for the destruction of the poison by oxydation. As soon as the heat of the summer returns, and is accompanied by a certain degree of moisture, that portion of the poison, as yet not destroyed by oxydation or dry heat, will commence to be vaporized, and, if breathed by any individual susceptible to the disease, will manifest its activity in the production of the phenomena characterizing yellow fever. The first case thus produced may recover without its true nature ever being suspected, while the disease may nevertheless be communicated to other persons, and thus have existed for some time until a severe case, characterized by unmistakable and prominent symptoms, such as black vomit, occur. Under such circumstances the tracing back of the disease to the first case generally proves a failure.

These facts will show that in the prevention of yellow fever our attention must be directed toward two chief objects, viz., the prevention of its fresh importation, and of its spreading after once having made its appearance, and, furthermore, the sure destruction of the poison generated by the cases arising.

The first object, of course, can only be attained by a properly arranged and conscientiously executed *quarantine*. So much has been said and written of late, both officially and publicly, about this subject—especially by those who claim its study as a specialty—as to require no additional remarks. Nevertheless, it may be said that the subject of quarantine has been presented as being much more complicated than it really is. Quarantine is quite an old institution, and has been practiced by many nations for many years. And it is more than certain that properly regulated and executed systems of quarantine do exist to-day, and, from all I have learned, even in our own country, which, moreover, might well serve as pattern for those stations along the coast of the Mexican Gulf, established for the purpose of preventing the importation of yellow fever. But though the administrative part of a quarantine may be quite simple, *as long as performed by intelligent and faithful officers*, the selection and application of a suitable disinfecting agent is more difficult; for, if this fails to destroy the yellow fever poison, when really present, the whole system of quarantine must appear à needless farce. The experiments made for the purpose of ascertaining the efficiency of a proper disinfectant must be made on matter, similar in nature to that representing the poison of contagious disease, and, as I have once hinted before in a short paper "On the Pathology of Yellow Fever"—published in the *New York Medical Journal*, Febr., 1879,—the fresh or dried lymph of the cow-pock-vesicle seems to be the most suitable for this investigation. A number of experiments of this kind have already been made by *Dougall*, *Baxter*, *Braidwood* and *Vacher*, all accompanied by nearly the same results. The summary of the results which Baxter obtained,—who extended his experiments even to the virus of infectious inflammation of guinea-pigs, and to glanders— is stated in his report as follows: *

1. "Evidence has been adduced to show that carbolic acid, sulphur dioxide, potassic permanganate, and chlorine, are all of them endowed with true disinfecting properties, though in very various degrees."

2. "It is essential to bear in mind that antiseptic is not synonymous with disinfective power, though, as regards the four agents enumerated above, the one is, in a certain limited sense, commensurate with the other."

3. "The effectual disinfectant operation of chlorine and potassic permanganate appears to depend far more on the nature of the medium through which the particles of infective matter are

---

***Reports* of the *Medical Officer of the Privy Council and Local Government Board.*—London, 1875.

distributed, than on the specific character of the particles themselves."

4. "When either of these agents is used to disinfect a virulent liquid containing much organic matter, or any compounds capable of uniting with chlorine, or of decomposing the permanganate, there is no security for the effectual fulfilment of disinfection short of the presence of free chlorine or undecomposed permanganate in the liquid after all chemical action has had time to subside."

5. "A virulent liquid cannot be regarded as certainly and completely disinfected by sulphur dioxide unless it has been rendered permanently and strongly acid. The greater solubility of this agent renders it preferable, *cœteris paribus*, to chlorine and carbolic acid, for the disinfection of liquid media."

6. "No virulent liquid can be considered disinfected by carbolic acid unless it contain at least two per cent. by weight of the pure acid."

7. "When disinfectants are mixed with a liquid, it is important to be sure that they are thoroughly incorporated with it; that no solid matters capable of shielding contagium from immediate contact with its destroyer be overlooked."

8. "Aërial disinfection, as commonly practiced in the sick-room, is either useless or positively objectionable, owing to the false sense of security it is calculated to produce. To make the air of a room smell strongly of carbolic acid by scattering carbolic powder about the floor, or of chlorine, by placing a tray of chloride of lime in a corner, is, so far as the destruction of specific contagia is concerned, an utterly futile proceeding."

9. "When aërial disinfection is resorted to, the probability that the virulent particles are shielded by an envelope of dried albuminous matter, should always be held before the mind. Chlorine and sulphur dioxide are, both of them, suitable agents for the purpose; the latter seems decidedly to be the more effectual of the two. The use of carbolic vapor should be abandoned, owing to the relative feebleness and uncertainty of its action. Whether chlorine or sulphur dioxide be chosen, it is desirable that the space to be disinfected should be kept saturated with the gas for a certain time, not less than an hour; and this in the absence of such gaseous compounds as might combine with or decompose the disinfectant, and so far impair its energy."

10. "When the thorough disinfection of a mass of solid or liquid matter, through which a contagium is disseminated, is impracticable, we should guard against giving a false security by the inadequate employment of artificial means. It is probable that all contagia disappear sooner or later under the influence of air and moisture, and that the absence of these influences may act as a preservative. When, therefore, we cannot advantageously or effectually supersede the natural process of decay, we must be sure that we do not hamper it by the injudicious use of antiseptics."

11. "Dry heat, when it can be applied, is probably the most efficient of all disinfectants. But, in the first place, we must be sure that the desired temperature is actually reached by every particle of matter included in the heated space; secondly, length of exposure and degree of heat should be regarded as mutually compensatory factors, within certain limits."

The above rules, laid down by Baxter for liquid or aërial disinfection, are very simple and will suffice for all purposes. In applying them, however, to the disinfection of ships, it will be found that a perfect disinfection of the whole cargo of a ship is almost impossible as long as the latter remains undisturbed, for the reason that the gaseous disinfectant cannot penetrate to its depth, or into the interior of the bales of merchandise, unless the ballast is broken. In order to thoroughly disinfect the latter, therefore, it must be broken, by unloading the ship and exposing it to the free atmosphere. Now, whether such a proceeding would be practicable, I am unable to decide, not being sufficiently acquainted with the commercial details and difficulties attending such an undertaking, I can only point to the fact, that unless every part of the cargo is exposed to the influence of the disinfectant or the atmosphere, any other mode of disinfection will be useless.

But, even, if the breaking of the ballast were practicable, a proper inquiry should first be made whether such a complicated labor is really necessary, that is, whether the noxious poison of yellow fever were, indeed, adhering to the merchandise of the cargo. Although I would not venture to deny the possibility of the poison being carried by the cargo of a ship hailing from the sea-ports of the West Indian Islands, or of the coast of South America, and consisting, as far as I know, generally of coffee, sugar, tobacco, etc.—articles which are generally kept in large warehouses—I cannot but think that such an occurrence must be very rare, unless these warehouses are situated in a densely populated locality where the yellow fever is raging, and the goods are handled by persons who have come into direct contact with yellow fever patients. And even, if this were the case, it appears that during or before the act of loading, the goods, generally lying along the wharves of the port, become sufficiently exposed to the atmosphere to render the vaporization and ultimate diffusion of the poison into the latter possible. It is, therefore, more probable that the poison is generally carried to a ship by the clothes of the sailors and passengers, as well as by the effects locked up in their trunks; and it is from these that the poison is communicated to the usually badly ventilated and confined spaces and cabins of the ship during the voyage. This supposition will appear more reasonable in considering the nature of those places and localities in a sea-port, generally visited by the sailors when on land.

If the view I have taken is correct, a thorough disinfection of the clothes and effects of the sailors and passengers, together with the cabins and general hold of the ship, would be all that is needed.

The disinfection of the ship may be effected in accordance with the general rules laid down by Baxter, together with the washing of the deck and other wood work. But as regards the clothes and effects of the passengers, the application of dry heat is preferable, as it is more reliable in its effects. This method of disinfection has been practiced for a number of years in European hospitals, both in England and Germany, and different apparatuses have been constructed for the purpose, the description of which may be found in the medical records. With the construction of the proper buildings and accommodations of a quarantine station, arrangements could be made that, while the clothes and other effects of the passengers are exposed to the dry heat of the disinfecting apparatus, they, themselves, might enjoy a good bath, for soap and water will prove, after all, the best disinfectant.

The degree of heat which woolen or other goods are able to bear without damage in the heated chamber during the process of disinfection has been determined by *Vallin.** He found that white blankets, if exposed to a temperature of 110° C. for two hours, slightly change in color, such as if they had been washed with hot water; if exposed to 115-120° for two hours, a slight yellowish coloration was noticed. Linen or cotton goods bear 115° very well, their color suffering only when exposed to 125° for two hours. The integrity of the goods becomes affected with a much higher (150°) temperature. Change in color occurs sooner with dry heat then when the heated air contains a large quantity of moisture. As regards the degree of heat required to destroy infectious matter, it appears that most germs of disease become innoxious with a temperature of 100° C., the septic matters, however, retain their power of infection even at this temperature.

The prevention of the yellow fever, after having been once introduced, or newly arisen—*events, possible in the face of the best sanitary regulations*,—appears to me no less important, and much more difficult, than to guard against its importation. Though it may be nearly impossible to extinguish the disease abruptly after it has once arisen, much can be done to prevent its spreading to a much greater extent, or from finally assuming the form of an epidemic. The only way by which this can be acccomplished is to prevent, or, at least, restrain the intercourse between the healthy and the sick. I have already pointed to the most pernicious custom of the female neighbors entering indiscriminately the house in which a patient lies sick of yellow fever, either to inquire after his welfare, or for idle curiosity. Now, though the first of these objects is very laudable, it must be dispensed with, when associated with the danger of carrying the poison to other houses and families, from which it may eventually spread over the same or other districts. Any one, who will reflect upon the complexity of the daily intercourse between the inhabitants of a large city, will become convinced of this fact. The first step, necessary to the prevention of the spreading of the disease, must therefore consist *in informing the people of the contagious nature of the disease, and showing them the danger of entering a house infected with the disease. There remains no doubt but that extensive epidemics have chiefly been due to the idea of the non-conta-*

* *Virchow u. Hirsh*, Jahresbericht f. d. Jahr 1877. vol. I. p. 507.

*giousness of yellow fever, prevailing among the people.* In advocating the strictest observance of non-intercourse with cases of yellow fever, I, of course, exclude those persons necessary to attend to the comfort and nursing of the patient. It is not my part to point out in this place the details of the manner in which this proposition may be carried out without too much inconvenience to the friends of the patient, but I am confident that with the proper system and precaution it can be done, and the disease be arrested, or kept in safe check until the colder weather sets in, when the fall of the temperature of the air will prevent the evaporation and diffusion of the poison.

Equally as important as the isolation of the cases is the complete disinfection or destruction of all objects to which the exhalations of the patient may have adhered during the course of the disease. Those articles with which the patient has come in immediate contact, such as bedding, shirts and other garments, should absolutely be burned, as soon as the patient is in convalescence, even if it should be done at the cost of the community, for it would be the cheapest mode of proceeding in the end. Such articles as will stand boiling and washing with soap should undergo the process, for, it may safely be guaranteed that the noxious poison, arriving in the gutters of the street after this procedure, will be surely deprived of its noxious properties; woolen articles may be disinfected by heat. As the poison adhering to the floors, doors and windows of the house is most effectually removed by plenty of soap and water, nothing would remain but the walls, which, if admissible, might be white-washed, or, if papered, thoroughly disinfected by one of the disinfectants in gaseous form, as recommended by Baxter.

From the above sketch it may be inferred that the prevention, or, at least, the checking of yellow fever, is not as difficult a task as might be supposed, if the proper precaution is observed, and the necessary means are conscientiously and systematically employed. A most important factor in the execution of these measures, however, will ever be the active co-operation of the inhabitants of the city themselves; the submission to some inconvenience by the individual for the welfare of the whole community, even at the sacrifice of a few individual rights.

